# A novel microtubule inhibitor promotes tumor ferroptosis by attenuating SLC7A11/GPX4 signaling

**DOI:** 10.1038/s41420-023-01713-6

**Published:** 2023-12-13

**Authors:** Nannan Ning, Ziqi Shang, Zhiping Liu, Zhizhou Xia, Yang Li, Ruibao Ren, Hongmei Wang, Yi Zhang

**Affiliations:** 1 Department of Clinical Laboratory, Qilu Hospital of Shandong University, Jinan, China; 2 Shandong Engineering Research Center of Biomarker and Artificial Intelligence Application, Jinan, China; 3 Center for Translational Medicine, The First Affiliated Hospital of Xi’an Jiaotong University, Xi’an, China; 4 Center of Intelligent Medical Engineering, School of Control Science and Engineering, Shandong University, Jinan, China; 5 Shanghai Institute of Hematology, National Research Center for Translational Medicine at Shanghai, Collaborative Innovation Center of Hematology, Ruijin Hospital, Shanghai Jiao Tong University School of Medicine, Shanghai, China; 6 Graduate School of Advanced Science and Engineering, Hiroshima University, Hiroshima, Japan; 7 International Center for Aging and Cancer, Department of Hematology of The First Affiliated Hospital, Hainan Medical University, Haikou, China; 8 Shanghai Institute of Hematology, State Key Laboratory for Medical Genomics, National Research Center for Translational Medicine, Ruijin Hospital affiliated to Shanghai Jiao Tong University School of Medicine, Shanghai, China; 9 Department of Pharmacology, School of Medicine, Southeast University, Nanjing, China

**Keywords:** Drug development, Cell death

## Abstract

MP-HJ-1b is a novel microtubule inhibitor that we designed and reported previously. Ferroptosis is a newly identified type of nonapoptotic cell death induced by ferrous catalysis and lipid peroxidation. Here, transcriptomics, proteomics, and molecular docking analyses were combined to explore the novel effects of MP-HJ-1b on tumors. Both omics analyses suggested that MP-HJ-1b affects ribosomes, and we confirmed that it inhibits the ribosomal component proteins RPL35 and MRPL28. Colchicine was used as an analog, and the results showed that MP-HJ-1b and colchicine increased reactive oxygen species and malondialdehyde levels and decreased reduced glutathione levels, suggesting that they promoted ferroptosis in HeLa cells. Specifically, MP-HJ-1b downregulated SLC7A11 and GPX4 to enhance the classical pathway of ferroptosis, while colchicine upregulated LC3A/B-II and enhanced autophagy. Clinically, the serum concentrations of ferrous ions, reduced glutathione, and Hcy were higher in cervical cancer patients than in healthy individuals. ALT, AST, Cho, HDL-C, and LDL-C levels were decreased in the serum of patients. Our study expands understanding of the way MP-HJ-1b promotes cell death and enriches research on microtubule inhibitors in the ferroptosis field.

## Introduction

Microtubule inhibitors play an important role in antitumor chemotherapy [1–4]. According to the binding site of the compounds on tubulin, these inhibitors are divided into three classes: taxol binding site inhibitors, vinca binding site inhibitors, and colchicine binding site inhibitors [5, 6]. In clinical applications, patients receiving paclitaxel and vinca alkaloids often develop drug resistance, so colchicine binding site inhibitors have become a new focus [7, 8]. MP-HJ-1b, a novel microtubule inhibitor we designed and reported previously, binds in the colchicine binding site [9]. MP-HJ-1b can promote microtubule depolymerization, block mitosis, and enhance apoptosis. It is certain that MP-HJ-1b can inhibit the proliferation of dozens of tumor cell lines. More importantly, MP-HJ-1b has overcome multidrug resistance in vitro and in vivo [9].

Ferroptosis is a novel form of programmed cell death caused by iron-dependent lipid peroxidation [10, 11]. Emerging evidence suggests the potential use of triggered ferroptosis for cancer therapy, particularly for the eradication of aggressive malignancies that are resistant to conventional therapies [12]. The induction of ferroptosis is mainly to inhibit cell membrane transporters and block intracellular antioxidant enzymes, and its iconic feature is the accumulation of lipid reactive oxygen species (ROS) in cells [11, 13, 14]. System Xc-, which consists consisting of SLC7A11 and SLC3A2, is a cystine/glutamate antiporter that transports cystine from the extracellular space to the intracellular space [12, 15]. Intracellular cystine can be reduced to cysteine, which is used to synthesize glutathione [11, 16]. As an antioxidant, glutathione peroxidase 4 (GPX4) uses glutathione as a substrate for the reduction of lipid hydroperoxides to lipid alcohols [11, 17]. Thus, the glutathione-GPX4 antioxidant system protects cells from ferroptosis, and the SLC7A11/GPX4 axis is the classical signaling pathway of ferroptosis [13]. Compared with normal cells, tumor cells appear to be more dependent on iron for growth, and drug-resistant tumor cells are more prone to ferroptosis, which provides a new research direction for cancer therapy [18–20].

Previous studies have shown that some microtubule inhibitors are dual-targeting compounds that also tend to inhibit kinases. For example, the colchicine binding site inhibitors XRP44X and combretastatin A4 can inhibit the RAS signaling pathway [21, 22]. Recently, several researchers have reported that microtubule inhibitors, such as paclitaxel and vinblastine, can also promote ferroptosis in tumor cells [23, 24]. The microtubule inhibitor MP-HJ-1b inhibits the proliferation of various tumor cells and overcomes multidrug resistance, but the mechanism of MP-HJ-1b has not been fully revealed. In this study, we used the cervical cancer (CCa) HeLa cell line as the experimental object, hoping to discover more effects of MP-HJ-1b on cell suppression and to provide support for research on microtubule inhibitors and CCa chemotherapy.

## Results

### Transcriptomic changes in HeLa cells treated with MP-HJ-1b

**Fig. 1:**
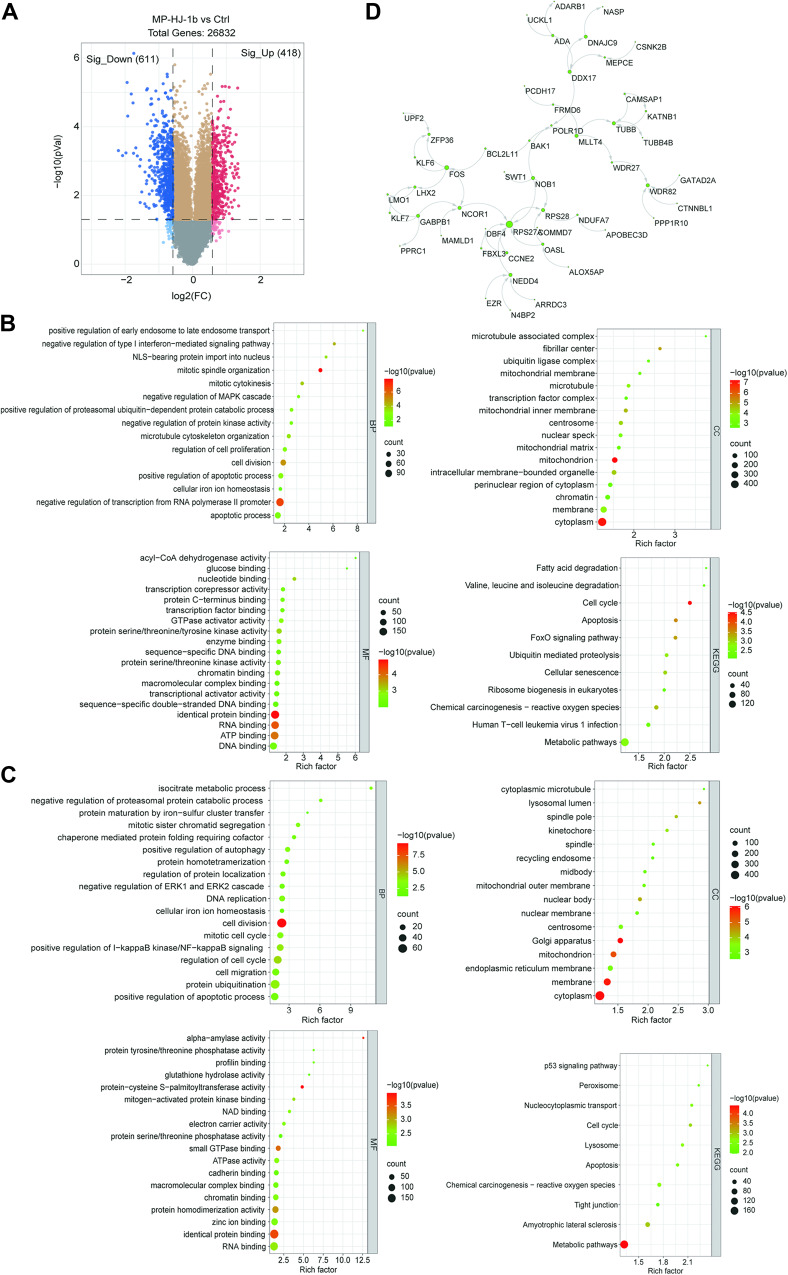
mRNA analysis of MP-HJ-1b in HeLa cells. **A** Volcano plot of the transcriptomic data (MP-HJ-1b *vs* Ctrl). Genes with *P* < 0.05 were considered differentially expressed genes (DEGs). **B** Significant GO terms and KEGG pathways related to genes upregulated by MP-HJ-1b stimulation. **C** Significant GO terms and KEGG pathways related to genes downregulated by MP-HJ-1b stimulation. **D** Gene interaction network of the top 50 hub differentially expressed genes (MP-HJ-1b *vs* Ctrl). The size of each gene node was determined by its frequency in the network, i.e., a larger node size means a higher frequency.

The microtubule inhibitor MP-HJ-1b has been shown to exert suppressive effects in many tumor cells. To explore additional functions of MP-HJ-1b, we treated HeLa cells with it and then assembled and analyzed transcriptomic data. Approximately 4% of 26832 genes detected were significantly regulated by MP-HJ-1b (Fig. 1A and Supplementary Excel S1). We performed Gene Ontology (GO) and Kyoto Encyclopedia of Genes and Genomes (KEGG) analyses of these differentially expressed genes (Fig. 1B, C). According to the enrichment analysis, genes upregulated by MP-HJ-1b treatment were associated mainly with identical protein binding, RNA binding, ATP binding (molecular function, MF), the cytoplasm, and mitochondria (cell component, CC) (Fig. 1B). Downregulated genes were enriched in cell division, protein ubiquitination (biological process, BP), the cytoplasm, the membrane and the *Golgi* apparatus (CC) (Fig. 1C). KEGG analysis showed that these differentially expressed genes were mostly concentrated in metabolic pathways (Fig. 1B, C). To further screen for the most significant hub genes, we uploaded these hub genes to the Cytoscape database for network analysis. The results indicated that MP-HJ-1b affects *FOS* (MF, DNA binding), *RPS27A*, *RPS28*, *WDR82* (WD repeat domain 82), and *TUBB* (Fig. 1D). *TUBB* refers to the tubulin β chain, which is clearly consistent with the inhibitory effect of microtubules. *RPS27A* and *RPS28* encode the constituent proteins of the 40S ribosome, suggesting that ribosomes might participate in the mechanism of action of MP-HJ-1b.

### Proteomic changes in HeLa cells induced by MP-HJ-1b

**Fig. 2:**
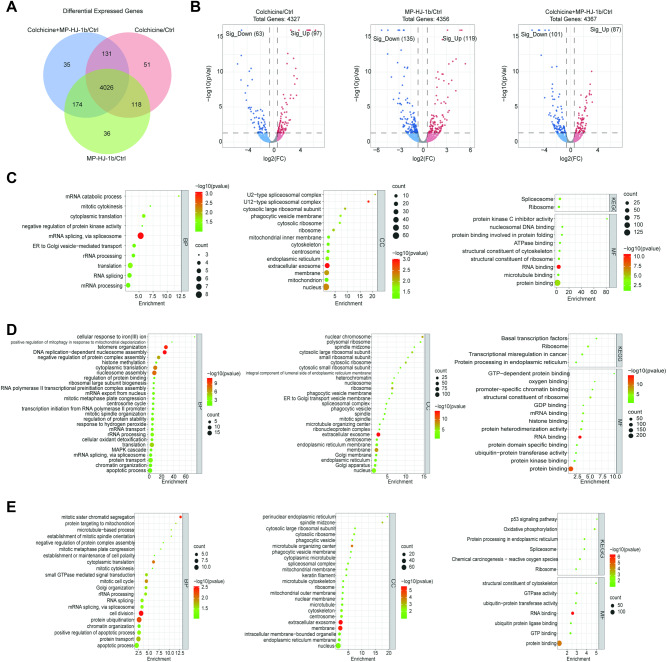
Protein analysis of MP-HJ-1b and colchicine in HeLa cells. **A** Venn diagrams showing the overlapping significantly regulated proteins the MP-HJ-1b/Ctrl, colchicine/Ctrl and MP-HJ-1b+colchicine/Ctrl groups. **B** Volcano plot showing protein expression levels (MP-HJ-1b/Ctrl, colchicine/Ctrl, MP-HJ-1b+colchicine/Ctrl), including those of upregulated and downregulated proteins in HeLa cells. **C**–**E** Upregulated and Downregulated GO enrichment and KEGG pathway terms among the MP-HJ-1b/Ctrl, colchicine/Ctrl and MP-HJ-1b+colchicine/Ctrl groups of HeLa cells.

To explore the new target of MP-HJ-1b, we used colchicine as an analog control to analyze the effect of MP-HJ-1b on proteins. Venn analysis showed that only a few proteins were specifically expressed in the three comparison groups, and most proteins were shared (Fig. 2A and Supplementary Excel S2). Compared with those in the control group, the expression levels of approximately 5% of proteins (160 in the colchicine group, 254 in the MP-HJ-1b group, 188 in the colchicine+MP-HJ-1b group) were significantly affected by the compounds (Fig. 2B). To functionally analyze the differences among these groups, we utilized GO terms and found that the differentially abundant proteins in all three comparison groups were enriched in extracellular exosomes in the CC category (Fig. 2C–E). The distinction was reflected mainly in the BP category: the differentially abundant proteins were significantly enriched in mRNA splicing in the colchicine/Ctrl group (Fig. 2C), associated with telomere organization and nucleosome assembly in the MP-HJ-1b/Ctrl group (Fig. 2D), and involved in cell division and protein ubiquitination in the colchicine and MP-HJ-1b cotreatment group (Fig. 2E).

**Fig. 3:**
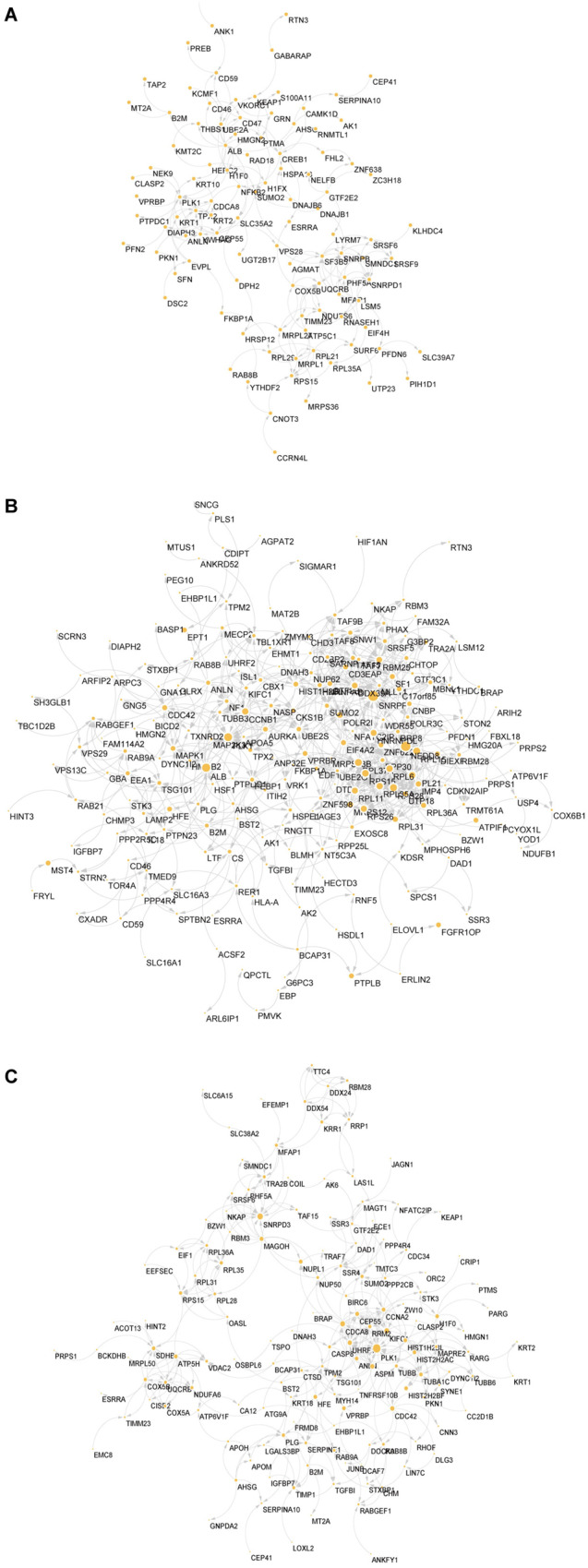
Protein interaction analysis of colchicine and MP-HJ-1b on HeLa cells. **A**–**C** Protein interaction analysis of differentially expressed proteins in three comparison groups (colchicine/Ctrl, MP-HJ-1b/Ctrl, and MP-HJ-1b+colchicine/Ctrl).

**Fig. 4:**
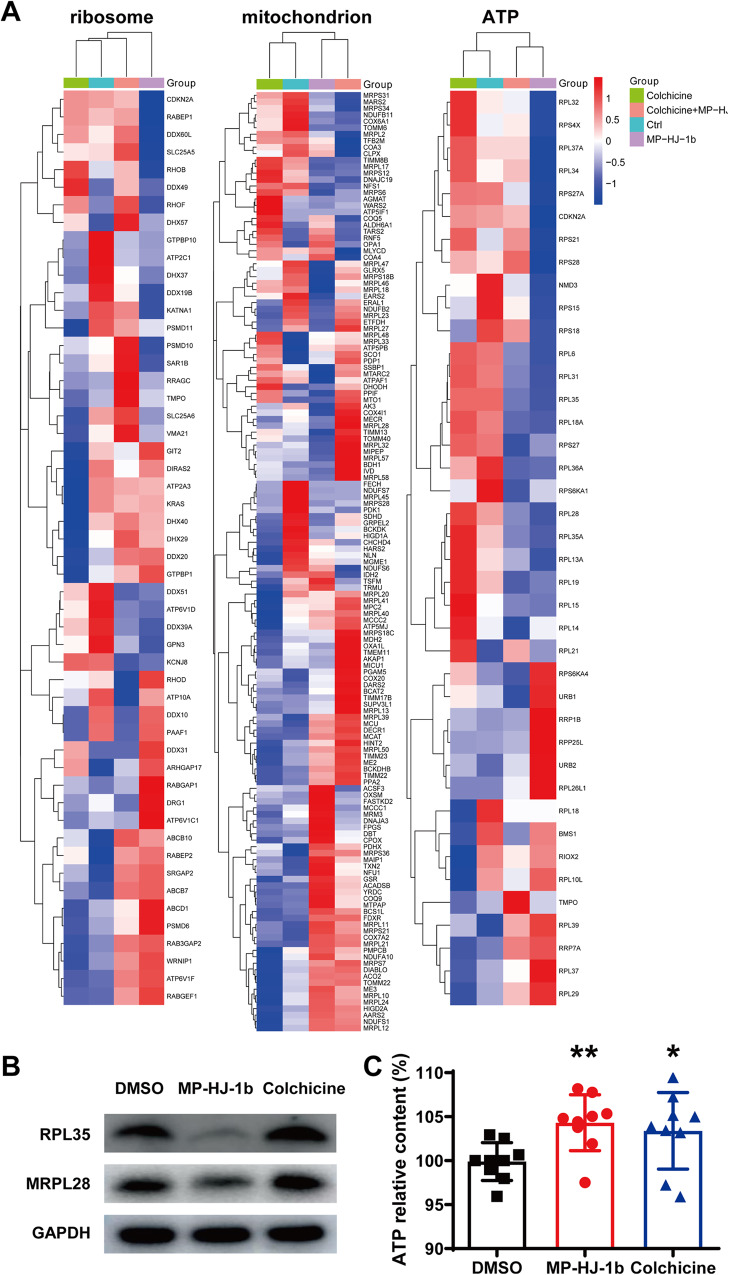
Functional analysis of MP-HJ-1b on HeLa cells. **A** Heatmap showing the differential expression of proteins related to ribosomes, mitochondria, and ATP with MP-HJ-1b and colchicine treatment. **B** Western blotting analysis of RPL35 (a ribosome protein) and MRPL28 (a mitoribosome protein). **C** Intracellular ATP measurement after 1 h of MP-HJ-1b and colchicine treatment. (*t* tests, *P < 0.05, **P < 0.01).

To further screen for the most significant hub proteins, we uploaded them to the STRING database for network analysis (Fig. 3A–C). The intersecting proteins in the colchicine/Ctrl group were PLK1, TPX2, CEP55, SF3B5, SRSF9, SNRPB, and SNRPD1, which are related to the cell cycle and mRNA splicing (Fig. 3A). The results of the MP-HJ-1b/Ctrl group involved ribosomes (RPL6, RPL11, RPL15, RPL35, RPL37, RPS15, RPS26, RPS28, MRPS12), the cell cycle (PLK1, CCNB1, NEDD8) and mRNA splicing (SNRPF) (Fig. 3B). The network of the colchicine+MP-HJ-1b/Ctrl group also included PLK1 and mRNA splicing protein (SNRPD3) (Fig. 3C). The changes induced by MP-HJ-1b compared to the other treatments were associated with ribosomes, consistent with the results of network analysis of the transcriptome. Therefore, we selected and analyzed the proteins related to ribosomes and energy metabolism and discovered that MP-HJ-1b significantly decreased the expression of many proteins (Fig. 4A). Furthermore, we performed western blotting experiments and found that MP-HJ-1b specifically decreased the expression of RPL35 (a ribosome protein) and MRPL28 (a mitoribosome protein) in HeLa cells (Fig. 4B). We also measured ATP by CellTiter-Glo Cell Viability Assay and found that MP-HJ-1b and colchicine were able to increase intracellular ATP at 1 h (Fig. 4C and Supplementary Fig. S1).

### MP-HJ-1b affects ferroptosis in HeLa cells

**Fig. 5:**
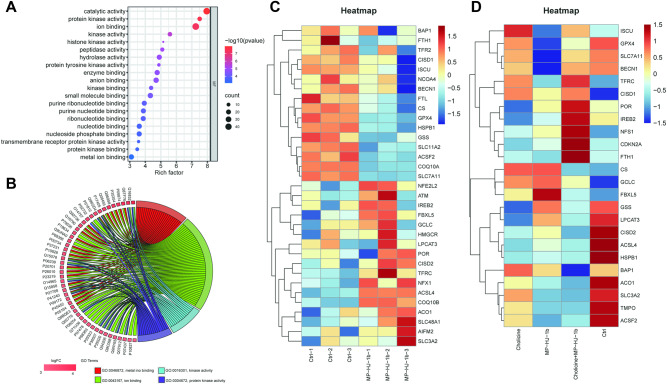
Proliferation is inhibited by regulating ferroptosis-related genes with MP-HJ-1b treatment in HeLa cells. **A** Molecular function of GO terms in molecular docking. **B** Chord diagram of GO terms for kinases and ion binding. **C** Heatmap showing the differential expression of genes related to ferroptosis with MP-HJ-1b treatment. **D** Heatmap showing the differential expression of proteins related to ferroptosis with MP-HJ-1b and colchicine treatment.

Although we identified the inhibitory effect of MP-HJ-1b on ribosomes, this was not sufficient to explain its novel action. Therefore, we performed molecular docking, and the first 6 candidates for MP-HJ-1b were DDR2, GSG2, PARP1, PTGS2, PLAU, and HDAC10, three of which were related to kinases (Supplementary Excel S3). We also assessed the GO terms on the top 50 proteins obtained by molecular docking and found that the genes were mainly involved in kinases and ion binding (Fig. 5A, B). First, we detected the effect of MP-HJ-1b on kinases and found that it increased the phosphorylation of many kinases (Supplementary Fig. S2), suggesting that MP-HJ-1b might not be a kinase inhibitor. Thus, we next explored ion binding. Considering the confirmed death-promoting effects of MP-HJ-1b on tumor cells and the correlation of ions with cell death, we focused on iron ions first. We selected and analyzed ferroptosis-related factors from the transcriptomic and proteomic data and found that MP-HJ-1b could alter their expression (Fig. 5C, D). In both data sets, ferroptosis suppressors such as GPX4, HSPB1, ISCU, and SLC7A11 were downregulated by MP-HJ-1b. Among ferroptosis drivers, ACO1, ACSL4, IREB2, and TFRC were upregulated in the transcriptomic data, and CS expression was increased at the protein level (Fig. 5C, D). This information suggested that MP-HJ-1b affected ferroptosis and might be associated with ferroptosis suppressors.

**Fig. 6:**
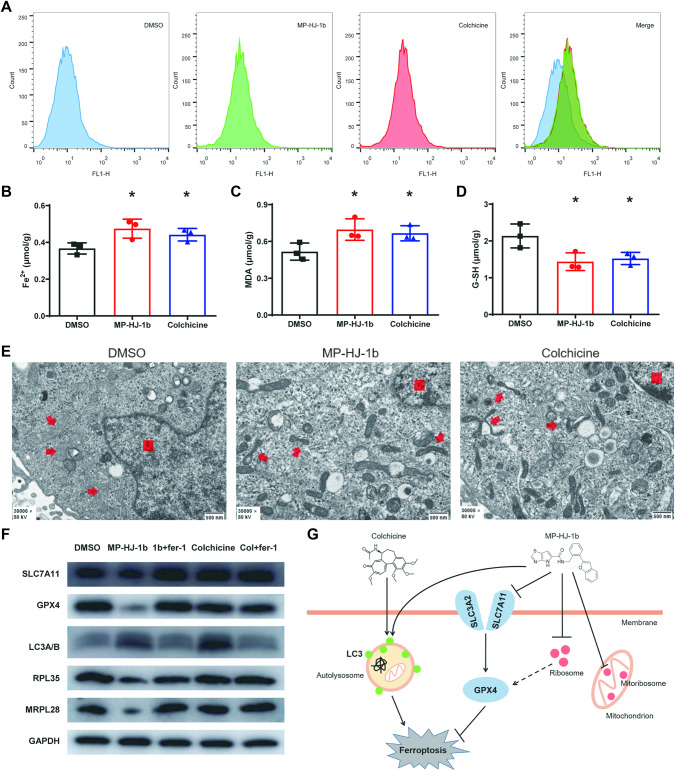
MP-HJ-1b and colchicine affect the ferroptosis pathway in HeLa cells. **A** Intracellular ROS measurement by flow cytometry after treatment with the two compounds. (Marker, BODIPY^TM^ 581/591 C11). **B** MP-HJ-1b and colchicine increase Fe^2+^ concentrations in HeLa cells. (*t* tests, *P < 0.05). **C** MP-HJ-1b and colchicine increase the concentration of MDA in HeLa cells. (*t* tests, *P < 0.05). **D** MP-HJ-1b and colchicine decrease intracellular G-SH levels. (*t* tests, *P < 0.05). **E** Electron micrograph. (magnification, 30000×; red arrow, mitochondrion; red rectangle, nucleus). **F** Western blotting analysis for SLC7A11, GPX4, LC3A/B, RPL35, and MRPL28. Ferrostatin-1 (fer-1) is a ferroptosis inhibitor. **G** Schematic of the mechanism by which the two compounds promote ferroptosis.

To test our speculation, the effects of a series of ferroptosis indicators were assessed in HeLa cells. MP-HJ-1b and colchicine promoted lipid peroxidation, increased the levels of ferrous ions and malondialdehyde (MDA) and decreased the level of reduced glutathione (G-SH; Fig. 6A–D). In addition, we observed the morphology of mitochondria and discovered that both compounds reduced mitochondrial volume and increased mitochondrial membrane density (Fig. 6E). These results suggested that MP-HJ-1b and colchicine promoted ferroptosis and that their similar binding sites on tubulin might be responsible. Surprisingly, western blotting revealed extremely different mechanisms of ferroptosis promotion between MP-HJ-1b and colchicine. MP-HJ-1b specifically reduced the expression of SLC7A11 and GPX4 and slightly upregulated LC3A/B-II, whereas colchicine did not alter SLC7A11 and GPX4 expression but significantly increased LC3A/B-II expression in cells (Fig. 6F and Supplementary Fig. S3 and S4). The results indicated that MP-HJ-1b promoted ferroptosis mainly through the classical SLC7A11/GPX4 pathway. Moreover, we also examined ribosomal proteins and found that ferrostatin-1 (Fer-1) could reverse the negative regulation of RPL35 and MRPL28 by MP-HJ-1b (Fig. 6F). Furthermore, we combined all the data and drew a schematic diagram of the mechanism by which MP-HJ-1b and colchicine promote ferroptosis (Fig. 6G).

### Analysis of CCa clinical samples

**Fig. 7:**
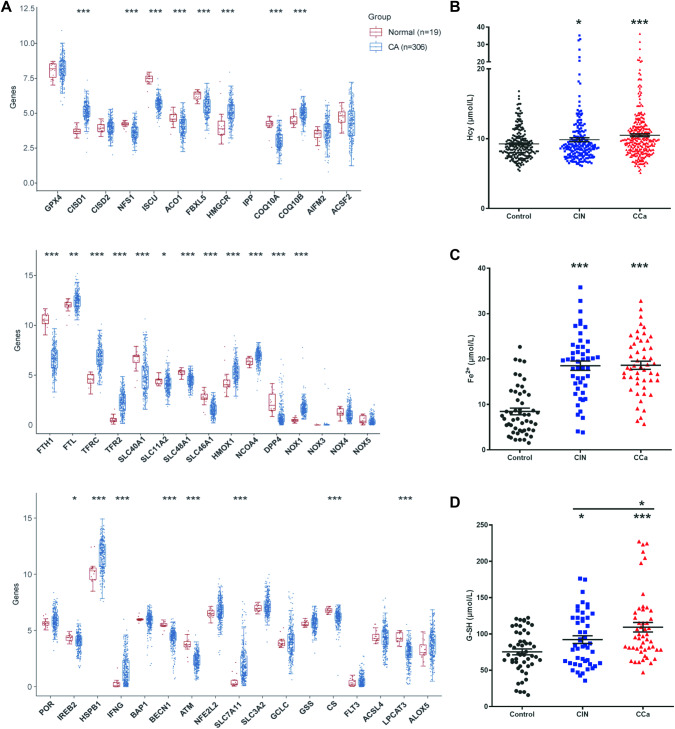
Analysis and detection of ferroptosis-related genes and serum indicators in human CCa. **A** Expression distribution of ferroptosis-related genes in human CCa tissues and normal tissues. Significant differences between two groups were identified by the Wilcoxon test; significant differences among the three groups were identified by Kruskal–Wallis test. Asterisks (*) indicate significance levels: *P < 0.05, **P < 0.01, ***P < 0.001. **B** Serum Hcy values in healthy, CIN and CCa patients. (*t* tests, *P < 0.05, ***P < 0.001). **C** Serum G-SH values in healthy control (*n* = 52), CIN (*n* = 48), and CCa (*n* = 50) patients. (*t* tests, *P < 0.05, ***P < 0.001). **D** Serum Fe^2+^ values in healthy control (*n* = 52), CIN (*n* = 48) and CCa (*n* = 50) patients. (*t* tests, ***P < 0.001).

CCa is a serious threat to women’s health, and the age of women diagnosed with CCa is decreasing. According to the TCGA database, the transcriptome of CCa is different from that of healthy individuals. We briefly analyzed the top 50 differentially expressed genes and found that proliferation-related genes were upregulated, while adhesion-related genes were downregulated (Supplementary Fig. S5). We selected and analyzed ferroptosis-related genes and found that several drivers (*ACO1*, *DPP4*, *BECN1*, *ATM*, *CS*, *LPCAT3*) were downregulated and some suppressors (*CISD1*, *HSPB1*, *SLC7A11*) were upregulated in CCa (Fig. 7A). In addition, we collected and analyzed the clinical biochemical data from CCa, cervical intraepithelial neoplasia (CIN) and healthy individuals, as listed in Table 1. Compared to CA-125, SCC seemed to be more suitable as a tumor marker, as its levels significantly differed among the CCa, CIN, and healthy groups. With regard to standard biochemical indices, the serum ALT, AST, Cho, and HDL-C levels of patients were lower than those of healthy individuals. In terms of ferroptosis, the level of Hcy, one of the raw materials for glutathione synthesis, was clearly increased in the CC group (Fig. 7B). Moreover, we collected serum from the patients and healthy individuals and measured G-SH and ferrous ions. Compared with healthy controls’ serum, patients’ serum contained higher G-SH and ferrous ion levels, and the concentration of G-SH was correlated with the progression of cancer (Fig. 7C, D).

**Table 1:** Clinical biochemical data of patients (CCa and CIN) and healthy controls.

Item	Control	CIN	*P* value	Control	CCa	*P* value	CIN	CCa	*P* value
	(*n* = 230)	(*n* = 205)		(*n* = 230)	(*n* = 311)		(*n* = 205)	(*n* = 311)	
SCC (ng/ml)	0.18 (0.12)	0.86 (0.64)	<0.001^a^	0.18 (0.12)	1.53 (3.64)	<0.001^a^	0.86 (0.64)	1.53 (3.64)	<0.001^a^
CA-125 (U/ml)	11.45 (6.21)	12 (8.1)	1.000^a^	11.45 (6.21)	13.2 (8.88)	<0.001^a^	12 (8.1)	13.2 (8.88)	0.002^a^
ALT (U/L)	13.95 (10.4)	12.3 (7.35)	0.055^a^	13.95 (10.4)	12.3 (9.2)	0.049^a^	12.3 (7.35)	12.3 (9.2)	1.000^a^
AST (U/L)	18.1 (6.025)	16.9 (5.9)	0.019^a^	18.1 (6.025)	16.7 (6.3)	0.002^a^	16.9 (5.9)	16.7 (6.3)	1.000^a^
GGT (U/L)	15 (8.25)	14 (7)	0.033^a^	15 (8.25)	15 (9)	1.000^a^	14 (7)	15 (9)	0.018^a^
Cho (mM)	5.04 ± 0.93	4.67 ± 0.89	<0.001	5.04 ± 0.93	4.65 ± 0.87	<0.001	4.67 ± 0.89	4.65 ± 0.87	0.767
HDL-C (mM)	1.46 (0.47)	1.34 (0.375)	0.001^a^	1.46 (0.47)	1.3 (0.35)	<0.001^a^	1.34 (0.375)	1.3 (0.35)	0.076^a^
LDL-C (mM)	2.83 (1.0325)	2.61 (1.055)	0.044^a^	2.83 (1.0325	2.66 (0.94)	0.082^a^	2.61 (1.055)	2.66 (0.94)	1.000^a^
TG (mM)	1.03 (0.84)	0.96 (0.615)	>0.050^a^	1.03 (0.84)	1.05 (0.68)	>0.050^a^	0.96 (0.615)	1.05 (0.68)	>0.050^a^

Normal distributed data were expressed as mean ± standard deviation, and non-normal distributed data were expressed as median (InterQuartile Range).

^a^Significance values adjusted for multiple tests with Bonferroni correction.

## Discussion

Recently, a great deal of effort has been expended to design and develop anticancer drugs based on ferroptosis induction. In this study, we found that MP-HJ-1b and colchicine reduced antioxidant capacity, increased lipid ROS accumulation and promoted ferroptosis. MP-HJ-1b specifically downregulated SLC7A11, GPX4, RPL35, and MRPL28, while colchicine significantly upregulated LC3A/B-II and enhanced autophagy.

Ferroptosis is a newly recognized form of cell death that was defined only a decade ago. In ferroptosis, intracellular iron accumulation and lipid peroxidation are key in triggering oxidative damage to cell membranes [10, 25]. Some microtubule inhibitors can induce ferroptosis, such as paclitaxel, which downregulates the glutathione-GPX4 pathway in ferroptosis metabolism [23, 24, 26]. We revealed that MP-HJ-1b, a novel microtubule inhibitor, can promote ferroptosis by downregulating SLC7A11 and GPX4. Following MP-HJ-1b or colchicine treatment, we detected a decrease in the G-SH level and increases in MDA and ROS levels, indicating that ferroptosis was triggered. Although previous studies have not clearly indicated that colchicine binding site inhibitors affect ferroptosis, it has been suggested that colchicine can regulate CYP2E1, which is associated with ferroptosis [27]. In addition, colchicine inhibits the degradation of autophagosomes by lysosomes, resulting in intracellular accumulation of LC3-II [28–30]. LC3-II/LC3-I is the classic activation marker of autophagy, which is associated with ferroptosis in tumor cells [31–34]. We observed that colchicine and MP-HJ-1b reduced the volume of the mitochondria and increased the mitochondrial membrane density, which is characteristic of ferroptosis. Colchicine markedly increased LC3-II level in HeLa cells, suggesting that the promotion of ferroptosis by colchicine might be mediated by autophagy. Moreover, ferrostatin-1(a ferroptosis inhibitor) reversed the inhibition of RPL35 and MRPL28 (the components of the 60S ribosome and 39S mitoribosome) by MP-HJ-1b. Ribosomes are frequently upregulated in tumor cells, and GPX4 is a selenoprotein whose synthesis is associated with the large subunit of ribosomes [35–38].

Previous reports have suggested that oxidative stress and antioxidants can assist in the diagnosis of CCa, and that the changes in parameter values can reflect responsiveness to treatment [39–41]. The levels of antioxidant substances such as glutathione and MDA are increased in various cancers, including CCa [39, 42–45]. Serum glutathione levels decrease with treatment, directly reflecting the responsiveness of cancer to chemoradiotherapy and predicting long-term control of cancer [40, 44, 46]. Ferroptosis-related molecules such as labile iron, ROS, and glutathione can be assessed, to monitor the ferroptosis process in vitro and in vivo. We found significantly higher serum G-SH concentrations in CCa patients than in healthy controls. Consistent with the effects of traditional chemoradiotherapy, MP-HJ-1b reduced the G-SH level in HeLa cell line. Our statistics showed that serum ALT, AST, Cho, HDL-C, and LDL-C levels were all lower in patients’ than in healthy controls. Ferrostatin-1 improves acute and chronic liver injury and reduces the levels of associated indicators ALT, AST, and TG [47–49]. In addition, previous research has shown that the expression of ferroptosis-related genes (RTN3, SLC25A1, and GPX4) in cardiovascular disease correlates with the serum HDL level [50].

## Conclusions

In this study, we found that MP-HJ-1b and colchicine promote ferroptosis and revealed MP-HJ-1b plays a novel role in ferroptosis by inhibiting the SLC7A11-GPX4 pathway. Our findings provide support for future studies on ferroptosis targeting and dual-targeted chemotherapeutic drugs.

## Materials and methods

### Clinical information and samples

We reviewed and transcribed the common clinical examination items of CCa patients (516 cases, including 205 cases of CIN) admitted and diagnosed in our hospital in 2021, and the corresponding data were obtained from the health examination population (230 cases). Leftover serum from healthy people’s (52 cases) and the patient’s (48 cases of CIN, 50 cases of CCa) admission examination was collected and frozen in a -80 °C freezer. The study was approved by the Ethics Committee of Qilu Hospital of Shandong University (No. KYLL-2019KS-210), and the written informed consent was obtained from each patient.

### Cell preparation for mRNA sequencing and protein profiling

HeLa cells (verified by STR profiling) were cultured in DMEM containing 10% fetal bovine serum. After treatment with DMSO or MP-HJ-1b (200 nM) for 12 h, the cells were collected in centrifuge tubes. The HeLa cells were mixed, reacted with TRIzol, and then sent to a company (CapitalBio Technology, Human Genome U133 Plus 2.0 of Affymetrix) for sequencing.

HeLa cells were cultured on the 100-mm diameter dishes (~1 × 10^7^ cells). Then, DMSO, MP-HJ-1b (200 nM), or colchicine (200 nM) was added, and the culture was continued for 12 h. The cells were collected, washed, and then sent to Shanghai Jiao Tong University for protein profiling.

### Transcriptomics and proteomics analysis

Transcripts with an absolute value of log2 (fold change) larger than 1 and *P* < 0.05 were considered differentially expressed. Proteins with *P* < 0.05 were considered differentially expressed. The function of individual genes was analyzed with the Database for Annotation, Visualization and Integrated Discovery (DAVID) annotation software. Using DAVID, we identified GO terms and KEGG pathways statistically overrepresented for the selected genes compared with the reference genes (all genes). The default settings were used for GO terms and KEGG pathways. Values of *P* < 0.05 were considered to indicate statistical significance.

### Western blotting

HeLa cells were cultured, treated with DMSO, MP-HJ-1b (200 nM), or colchicine (200 nM) for 12 h, or treated with ferrostatin-1 (2 µM) for 6 h, and then treated with MP-HJ-1b or colchicine for 12 h. The cells were harvested and lysed in RIPA lysis buffer with protease inhibitors and phosphatase inhibitors. The proteins were run in a 10% SDS-PAGE gel and transferred to a PVDF membrane. The PVDF membrane was blocked and incubated with primary antibody overnight at 4 °C. After incubation with the secondary antibody, the membrane was exposed to ECL and imaged.

The specific primary antibodies were against the following proteins: GAPDH (Cell Signaling Technology, 2118), RPL35 (Abcam, ab190162), MRPL28 (Abcam, ab196842), SLC7A11 (Cell Signaling Technology, 12691 s), GPX4 (Abcam, ab125066), and LC3A/B (Cell Signaling Technology, 4108).

### ATP level assay

HeLa cells were cultured, collected, counted, and seeded into a 96-well plate (20,000 cells per well). DMSO, MP-HJ-1b (200 nM), or colchicine (200 nM) was added to the cells, and the cells were incubated in an incubator for 1 h. CellTiter-Glo Reagent (Promega, G7572) was added to each well, and the 96-well plate was incubated at room temperature for 10 min. Then ATP was measured using the lum mode of the microplate reader.

### Molecular docking

We developed a multichannel deep neural network for predicting the drug-protein affinity, and the model was trained on the DrugBank 5.0 database. The input of the model included the representations of protein and drug, and the output was the predicted drug-protein affinity. Two channels were fed the protein representations (i.e., one hot encoding of protein sequence, K-mer features of protein sequence), and the other two channels were fed the drug representations (i.e., extended-connectivity fingerprints (ECFP), molecular graph of the drug). We screened the targets of MP-HJ-1b with a well-trained drug-target interaction prediction system and listed the top-ranked candidates.

### Lipid peroxidation assay

HeLa cells were treated with DMSO, MP-HJ-1b (200 nM), or colchicine (200 nM) for 12 h, and then collected and washed. The cells were mixed with BODIPY^TM^ 581/591 C11 (Thermo Fisher Scientific, D3861) and incubated at 37 °C for 30 min. After washing with PBS buffer, these cells were analyzed for ROS by flow cytometry (Becton, Dickinson and Company, FACS Calibur).

### Fe^2+^ ELISA (Elabscience, E-BC-K773-M)

HeLa cells were centrifuged and collected after DMSO, MP-HJ-1b (200 nM), or colchicine (200 nM) treatment for 12 h. The cells were resuspended in buffer agent and disrupted by sonication, and the supernatant was collected after centrifugation at 10,000×*g*. The supernatant was mixed with chromogenic agent and incubated at 37 °C for 10 min. After centrifugation, the supernatant was added to a 96-well plate, and the OD values were measured at 593 nm with a microplate reader (BioTek, SynergyH1).

In all, 60 µl human serum was mixed with 180 μl buffer solution, and centrifuged at 5000×*g* for 5 min. 200 µl supernatant was added to a 96-well plate, and 100 µl chromogenic agent was dropped into the same well. After incubation at 37 °C for 10 min, the OD values were measured at 593 nm using a microplate reader.

### MDA ELISA (Nanjing Jiancheng Bioengineering Institute, A003-2)

After incubating HeLa cells with DMSO, MP-HJ-1b (200 nM), or colchicine (200 nM) for 12 h, the cells were harvested in 250 µl PBS buffer and sonicated. The homogenate was centrifuged, and 200 μl supernatant was taken out and mixed with 200 μl Reagent I, 3 ml Reagent II, and 1 ml Reagent III. The mixture was then placed in a 95 °C water bath for 40 min. The samples were cooled and placed in a microplate reader, and the OD values were measured at 532 nm.

### G-SH ELISA (Nanjing Jiancheng Bioengineering Institute, A006-2-1)

HeLa cells were stimulated with DMSO, MP-HJ-1b (200 nM), or colchicine (200 nM) for 12 h, collected in 150 µl PBS buffer and sonicated. 100 μl homogenate was mixed with 100 μl precipitating agent, and the mixture was centrifuged at 3500 rpm for 10 min. 100 μl supernatant was added to a 96-well plate, and then buffer agent and chromogenic agent were added to the same well. After the reaction was allowed to proceed for 5 min at room temperature, the OD values were measured at 405 nm with a microplate reader.

In total, 50 µl human serum was mixed with 200 μl precipitating agent, and the mixture was centrifuged at 3500 rpm for 10 min. The supernatant was added to a 96-well plate, and the following steps were the same as above.

### Cell preparation for transmission electron microscopy

HeLa cells were cultured in dishes and treated with DMSO, MP-HJ-1b (200 nM), or colchicine (200 nM) for 12 h. The medium was removed and the cells were washed twice with PBS buffer. The cells were stripped with a scraper and harvested by centrifugation at 2000 rpm for 5 min. The cells were washed again with PBS buffer and collected by centrifugation at 2000 rpm for 10 min. The supernatant was removed, and 2.5% glutaraldehyde fixation solution was slowly added dropwise. The samples were then sent to the electron microscopy laboratory.

### Supplementary information

MP-HJ-1b-ferroptosis_supplementary

Original western blots

Supplementary Excel S1

Supplementary Excel S2

Supplementary Excel S3

## Data Availability

All data are provided and displayed in the manuscript and supplementary materials.
