# Supplementary material for: A novel microtubule inhibitor promotes tumor ferroptosis by attenuating SLC7A11/GPX4 signaling
Source: Cell Death Discov. 2023 Dec 13;9:453. doi: 10.1038/s41420-023-01713-6 (PMC10716160; doi:10.1038/s41420-023-01713-6)
Supplement: Supplementary file 1 — MP-HJ-1b-ferroptosis_supplementary [file 41420_2023_1713_MOESM1_ESM.docx]

**Supplementary Information**

**Supplemental tables (Three excel files)**

**Supplementary Excel S1. MP-HJ-1b affects transcriptomic in HeLa cells.** Sequencing data of intracellular mRNA after DMSO and MP-HJ-1b treatment.

**Supplementary Excel S2. MP-HJ-1b affects proteomic in HeLa cells.** Mass spectrometric data of intracellular proteins after DMSO, colchicine, MP-HJ-1b and colchicine+ MP-HJ-1b treatment.

**Supplementary Excel S3. 100 potential candidate targets of MP-HJ-1b.** Based on molecular docking technology and compound-protein affinity, the possible target of paclitaxel was predicted. The target 83 was tubulin, which was a confirmed target for MP-HJ1b.

**Supplemental figures**


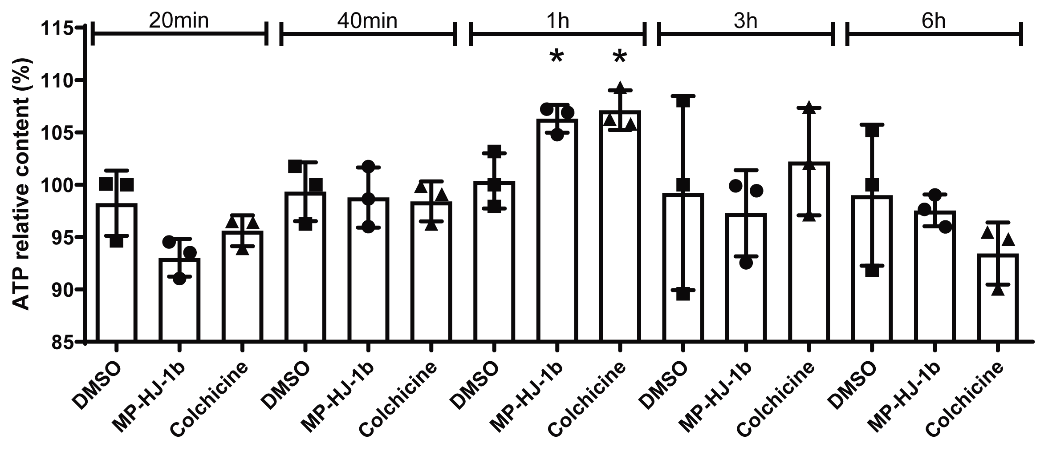


**Figure S1. MP-HJ-1b affects ATP in HeLa cells.** Intracellular ATP measurement after MP-HJ-1b and colchicine treatment. (*t*-tests, **P*<0.05)


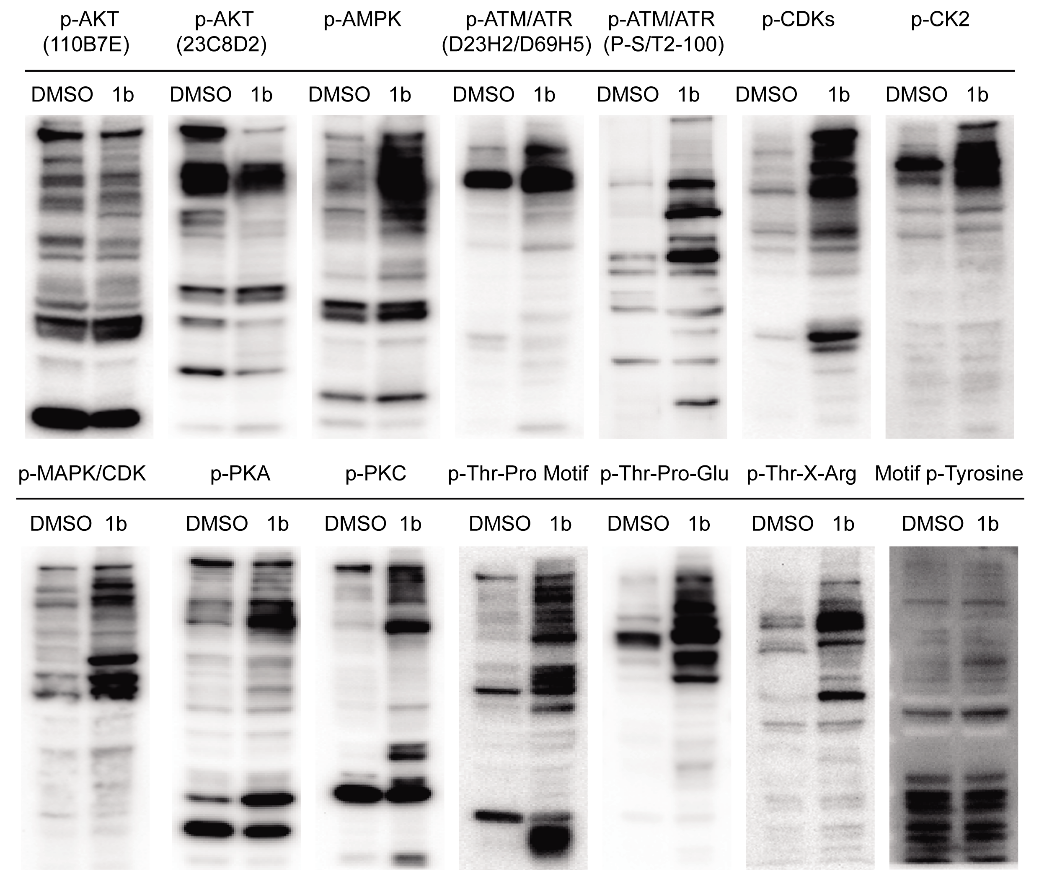


**Figure S2. MP-HJ-1b affects the phosphorylation of kinases in HeLa cells.** Western blotting analysis for multiple kinases. Primary antibodies belong to the kinomeView profiling kit (Cell Signaling Technology, 9812) and do not target a specific molecule, so the bands are not single.


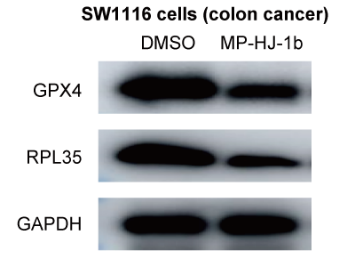


**Figure S3. MP-HJ-1b affects ferroptosis and ribosome in SW1116 cells.** Western blotting analysis for GPX4 (ferroptosis pathway) and RPL35 (60S ribosome). SW1116 is a colon cancer cell line.


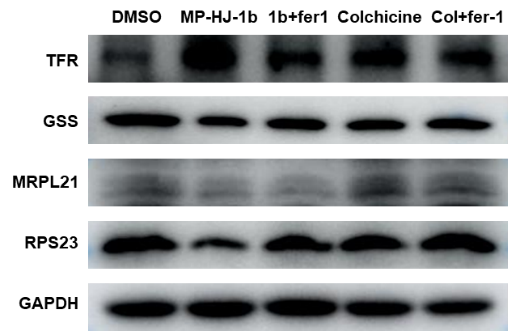


**Figure S4. MP-HJ-1b affects ferroptosis and ribosome in HeLa cells.** Western blotting analysis for TFR (transferrin receptor), GSS (glutathione synthetase), MRPL21 (a mitoribosome protein) and RPS23 (a ribosome protein).


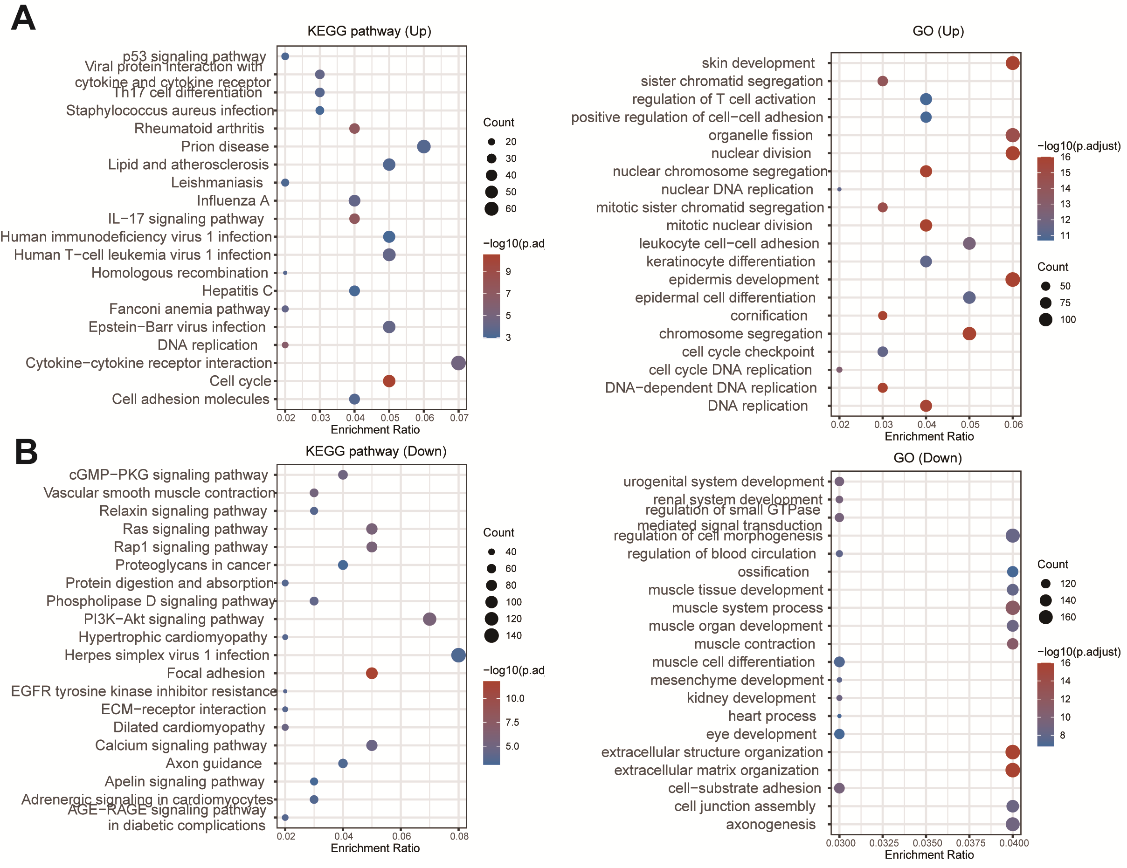


**Figure S5. Differentially expressed mRNA analysis of cervical cancer and healthy control.** KEGG and GO enrichment analysis of the top 50 differentially expressed genes in cervical cancer and healthy control tissues.

**Supplemental methods**

**ATP level assay**

HeLa cells were seeded into a 96-well plate (20,000 cells per well) and treated with DMSO, MP-HJ-1b or colchicine. After 20 minutes, 40 minutes, 1 hour, 3 hours or 6 hours of incubation, the cells were ruptured with CellTiter-Glo Reagent (Promega, G7572), then the ATP values were measured with a microplate reader.

**Western blotting**

The western blotting experiments were performed as described previously. The specific primary antibodies are as follows: TFR (Santa Cruz, sc-32272), GSS (Santa Cruz, sc-166882), MRPL21 (Abbkine, ABP51836), RPS23 (Santa Cruz, sc-100837) and kinomeView profiling kit (Cell Signaling Technology, 9812).

The primary antibodies in kinomeView profiling kit: p-Akt substrate (110B7E), p-Akt substrate (23C8D2), p-(Ser/Thr) AMPK substrate (P-S/T^2^-102), p-(Ser) ATM/ATR substrate (D23H2/D69H5), p-(Ser/Thr) ATM/ATR substrate (S*/T*QG) (P-S/T^2^-100), p-(Ser) CDKs substrate, p-(Ser/Thr) CK2 substrate (P-S/T^3^-100), p-MAPK/CDK substrate (34B2), p-PKA substrate (100G7E), p-(Ser) PKC substrate (P-S^3^-101)， p-Thr-Pro motif antibody, p-Thr-Pro-Glu (C32G12), p-Thr-X-Arg motif antibody, p-Tyrosine (P-Tyr-1000).

SW1116 cells were cultured and treated with MP-HJ-1b for 12 hours. The cells were collected and lysed in RIPA Lysis Buffer. And the subsequent steps were the same as previously described.
