# Supplementary figures and images for: A novel microtubule inhibitor promotes tumor ferroptosis by attenuating SLC7A11/GPX4 signaling
Source: Cell Death Discov. 2023 Dec 13;9:453. doi: 10.1038/s41420-023-01713-6 (PMC10716160; doi:10.1038/s41420-023-01713-6)

**Figure 4B**

RPL35

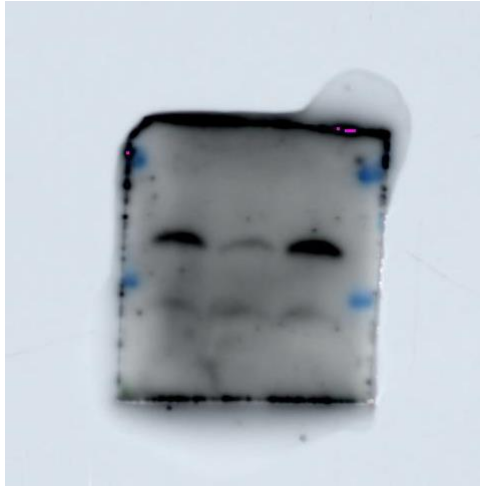

MRPL28

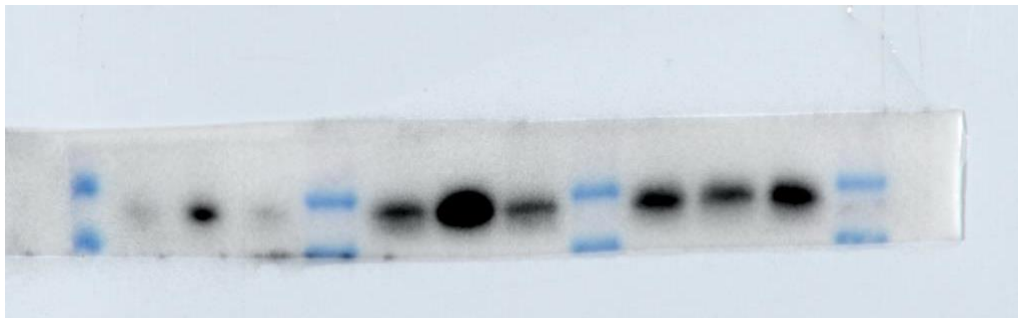

GAPDH

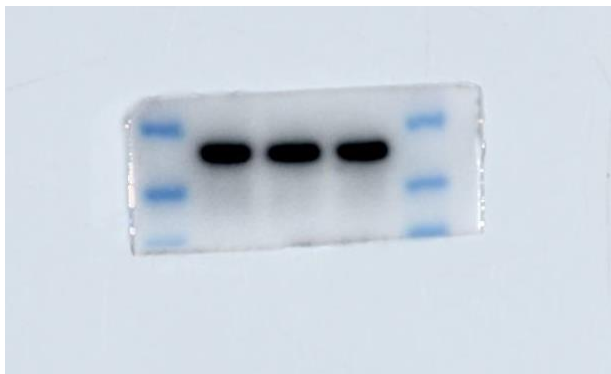

**Figure 6F**

SLC7A11

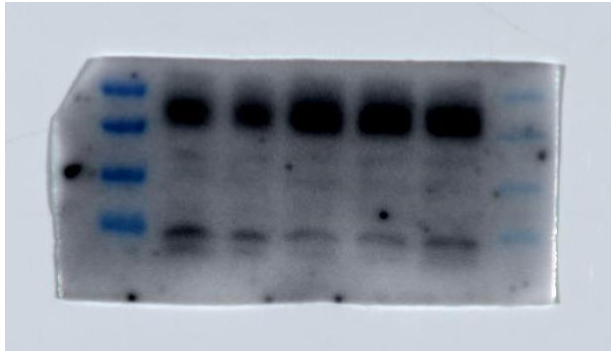

GPX4

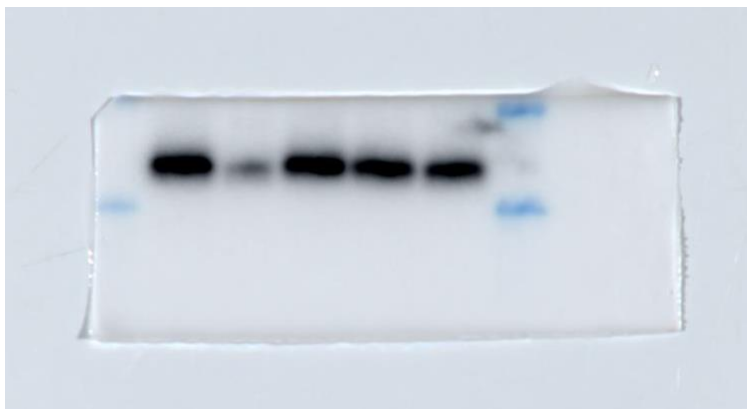

LC3A/B

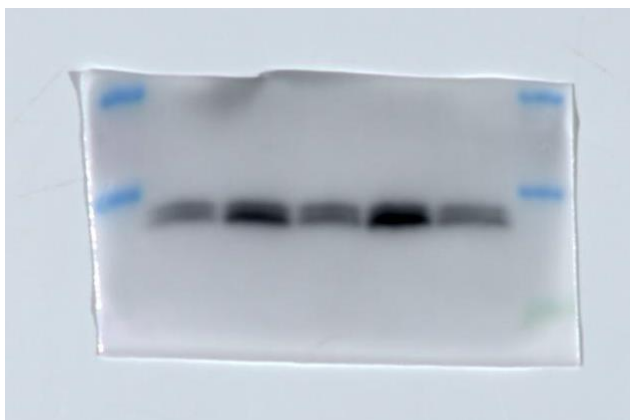

RPL35

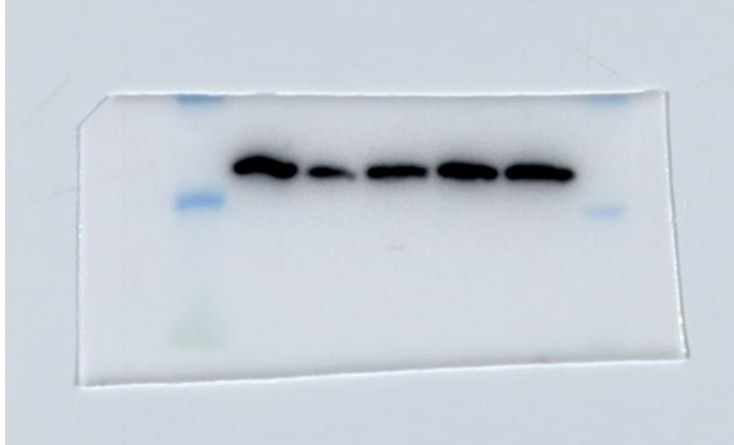

MRPL28

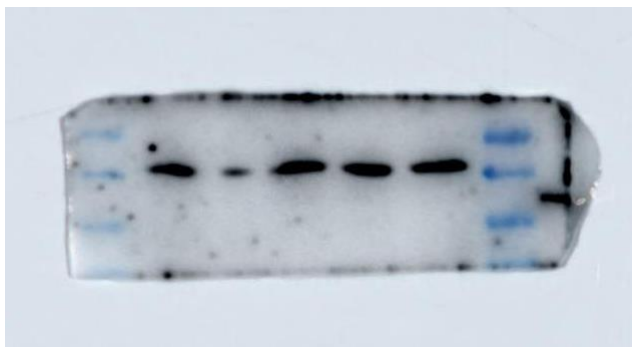

GAPDH

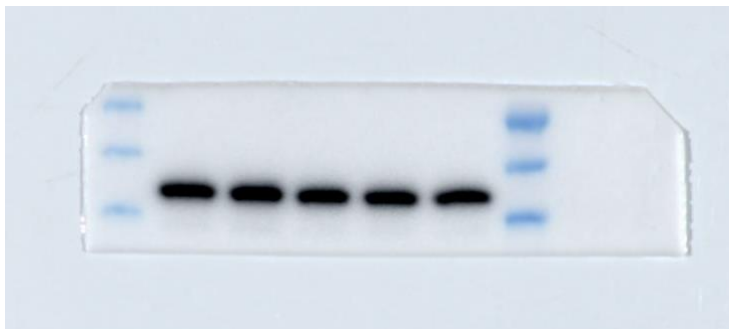

Supplement: Supplementary file 2 — Original western blots [file 41420_2023_1713_MOESM2_ESM.pdf]
